# Evaluation of the Practice of Performing Abdominal CT Scan with Manually Administered Fixed Dose of Contrast in Achieving Adequate Hepatic Enhancement: An Institutional-Based Cross-Sectional Study

**DOI:** 10.1155/2023/9563310

**Published:** 2023-02-21

**Authors:** Tesfaye Kebede Legesse, Mekdelawit Mengistu Getaneh, Semira Abrar Issa

**Affiliations:** Addis Ababa University, College of Health Sciences, Department of Radiology, Addis Ababa, Ethiopia

## Abstract

**Background:**

Intravenous (IV) iodine-based contrast agents are administered during computed tomography (CT) examination to enhance the density differences between lesions and surrounding parenchyma, which is important for lesion characterization, and to demonstrate vascular anatomy and vessel patency. Quality of contrast enhancement has a significant influence on diagnostic interpretation and subsequent management. In this study, we assessed the quality of portal venous phase abdominal CT scans performed with a manual injection of a fixed dose of contrast, which is the routine practice at Tikur Anbessa Specialized Hospital (TASH). The effect of age and sex was also assessed.

**Method:**

A hospital-based retrospective review was performed to identify patients who have had a precontrast and postcontrast abdominal CT scan from November 4, 2020, to September 30, 2022. All patients with abdominal CT scans having precontrast and portal venous phase scans were included in the study. All CT scans were reviewed by the principal investigator and the quality of contrast enhancement was assessed.

**Results:**

In this study, there were a total of 379 patients. The mean hepatic attenuations in precontrast and portal venous phase scans were 59.05 ± 6.69 HU and 103.73 ± 12.84 HU. The proportion of scans with less than 50 HU enhancement was 68% (*n* = 258). There was a significant association between age and sex with contrast enhancement.

**Conclusion:**

The hepatic contrast enhancement pattern of abdominal CT scan at the study institution reveals a concerning degree of image quality. This is evidenced by the high number of suboptimal contrast enhancement indices and the highly variable enhancement patterns across different patients. This can have a negative impact on the diagnostic performance of CT imaging and can adversely affect the management. Furthermore, both sex and age affect the pattern of enhancement.

## 1. Introduction

Multidetector CT (MDCT) is the workhorse for hepatic imaging as it demonstrated excellent spatial and contrast resolution added with speed and reproducibility. The other advantage of MDCT is the use of intravenous (IV) contrast agents to enhance the diagnostic capacity and the ability to postprocess images into multiplanar formats [1]. MDCT provides excellent morphologic information about both the disease and its relationship to normal anatomy [1].

Because of the dual blood supply to the liver, the organ can be imaged during multiple phases [1]. This is achieved through the administration of intravenous iodine-based contrast agents [2]. Contrast agents used during CT scans are useful to enhance density differences between lesions and the surrounding parenchyma, to demonstrate vascular anatomy and vessel patency, and to characterize lesions by their contrast enhancement patterns [2].

Four phases of liver parenchyma attenuation can be distinguished after contrast material injection, which includes the following: (1) hepatic arterial phase, (2) portal venous phase, (3) parenchymal phase, and (4) delayed or equilibrium phase depending on the time taken from the injection of contrast to scanning [3]. Most of the abdominal CT scan is performed during the precontrast and portal venous phase to decrease the radiation dose from repeated scans in different phases unless specifically justified for some clinical indications [4].

Unenhanced CT is useful for the evaluation of depositional diseases (e.g., hepatic steatosis and hemochromatosis), liver calcifications, haemorrhage, and some high-contrast embolic materials used for therapeutic procedures [2].

Maximum enhancement of the liver parenchyma is attained during the portal venous phase to demonstrate hypovascular lesions as low attenuation masses on the background of a brightly enhanced liver parenchyma. Maximum enhancement of the liver parenchyma occurs at 60 to 120 seconds, following hepatic arterial enhancement as two-thirds of the hepatic blood supply comes from the portal vein, allowing time for contrast to pass through the spleen and gastrointestinal tract into the portal veins [2].

Contrast administration and the timing of CT scan must be carefully planned to optimize the differences in enhancement patterns between the lesions and normal tissues. The density of the unenhanced normal liver at CT typically ranges between 55 and 65 HU [1]. Liver parenchyma is at its peak enhancement with a density >110 HU (an increase of at least 50 HU from the unenhanced baseline) during the portal venous phase [3, 5].

Adequacy of CT contrast enhancement has a significant influence during image interpretation, which also has an implication on the management. This is affected by numerous interacting factors which are contrast-, patient-, or machine-related. One of the critical contrast-related factors that determine the quality of the CT image is the mode of contrast injection, which affects the contrast injection duration, which is defined as the time from the beginning to the completion of the injection of contrast [6, 7].

Another important factor is the contrast biodistribution into the vascular spaces, which is related to the body size. As a consequence, if a fixed amount of iodine is administered to patients, some may receive an unnecessary high dose while others may receive a suboptimal dose [8]. Patient's age and sex also affect the adequacy of contrast enhancement [8]. The American College of Radiology (ACR) recommends utilizing an automated power injection device with nonionic contrast material in a dose of 572 mg/kg [9, 10].

In our institution, we use a fixed dose of 100 ml of nonionic contrast material (350–370 mg/ml) administered using the traditional (manual) mode of contrast injection. Despite this, there are not enough studies regarding the image quality of abdominal CT performed with such setup. This study aims to assess the quality of liver enhancement in portal venous phase scans performed with manual injection at a fixed dose of contrast and to evaluate the effect of gender and age on liver enhancement.

## 2. Materials and Methods

### 2.1. Study Area and Setting

The study was conducted at Tikur Anbessa Specialized Hospital (TASH), which is the largest tertiary referral hospital in Ethiopia, where CT was performed on a 128-slice-MDCT scanner (Philips computed tomography). The routine practice in the CT scanning procedure is as follows. Before scanning, the patients were instructed to fast for two to four hours. Most of the scanning was performed with preset peak tube voltage and tube current. First, unenhanced images were acquired then followed by a fixed dose of 100 ml of nonionic contrast material (350–370 mg/ml) which was administered using the traditional (manual) mode of contrast injection (as automatic contrast injectors are unavailable). After postprocessing, the image is sent to PACS (picture archiving and communication software) Medweb software.

### 2.2. Study Design and Study Population

An institutional-based retrospective cross-sectional study was conducted to evaluate the quality of liver enhancement in the portal venous phase on abdominal CT scans taken from November 4, 2020, to September 30, 2022. The source populations were all patients who have had abdominal CT scan imaging. The study populations were all patients who had precontrast and portal venous phase abdominal CT scan imaging. Those patients with IVC thrombosis, hepatic vein thrombosis and portal venous thrombosis, and cirrhotic liver and patients with two adjoining liver segment lesions were excluded.

### 2.3. Sample Size Determination

The sample size is calculated based on infinite population formula with the following parameters; *P* value as 50% (0.5), Z (standard score) = 1.96, CI (confidence interval) = 95%, and D (margin of error) = 5% (0.05). Based on this formula, the calculated sample size was 384.

The sample size was determined by using the following formula:

(1) $$\begin{array}{c} N=Z2P\frac{1-P}{d2}\text{,} \end{array}$$

where *n* = sample size, *Z* = statistic for a level of confidence, *PP* = expected prevalence or proportion, and *d* = margin of error, which corresponds to the level of precision of results desired.

(2) $$\begin{array}{c} n=\left(1.96\right){}^{2}0.5\frac{\left(1-0.5\right)}{0.05{}^{2}}\text{,}\\ n=384.16\text{.} \end{array}$$

### 2.4. Data Collection Procedure

**Figure 1:**
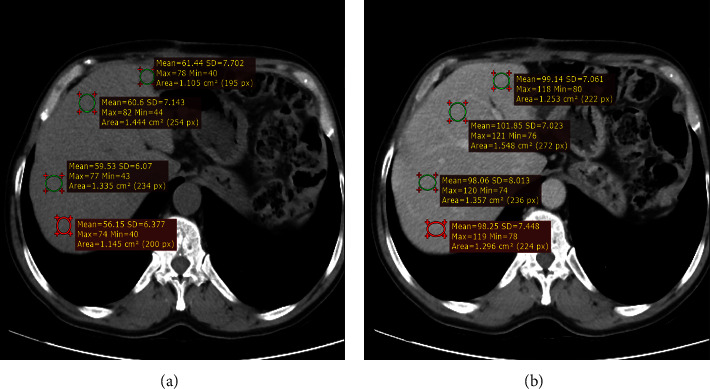
Axial precontrast (a) and postcontrast portal venous phase (b); axial CT scans at the same level taken in TASH, 2022 G.C, show mean hepatic attenuation at the four different locations of measurement.

A data collection tool was developed and patients who had precontrast and postcontrast abdominal CT scans were traced from PACS (Medweb) by using the patient medical record number (MRN). The mean CT values in Hounsefield (HU) units were measured in the right anterior section of the liver, right posterior section of the liver, left lateral section of the liver, and left medial section of the liver for all patients on the CT console monitor using a circular region of interest (ROI) cursor, ranging in size from 1 cm^2^ to 2 cm^2^ in diameter on unenhanced and portal phase images, and then were averaged (Figure 1). Blood vessels and bile ducts were excluded from all measurement areas.

Quantitative degrees of contrast enhancement were expressed as contrast enhancement indices (CEIs), which were calculated by subtracting the average CT values on unenhanced images from those of the contrast-enhanced images.

The data were collected by the principal investigator strictly following the procedures consistently for all study populations. The data collection tool was checked thoroughly for its completeness before starting the data collection.

### 2.5. Data Analysis and Interpretation

Data entering, coding, and cleaning were performed using Microsoft Excel and later were exported to SPSS version 26 for analysis. Statistical analysis was performed with the aid of SPSS statistical software. Means and ranges were calculated for continuous variables. An independent *t*-test was performed to determine the effect of patient's sex and age on contrast enhancement. A value of *P* < 0.05 was considered statistically significant.

## 3. Results

**Figure 2:**
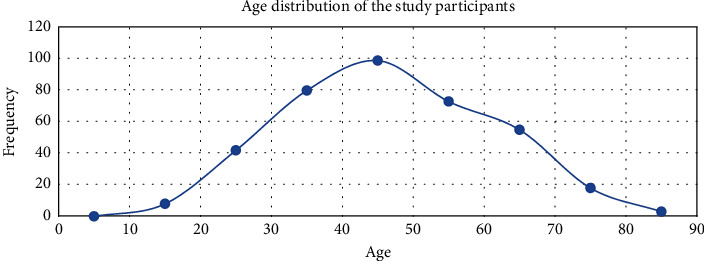
Age distribution of patients with precontrast and portal venous phase abdominal CT scan imaging at TASH, 2020–2022 G.C.

In this study, a total of 379 patients were included. The ages ranged from 15 to 85 years with a mean of 47.85 ± 14.82 years (Figure 2). Female to male ratio was 1.28 : 1 with females representing 56.2% (*n* = 213) of the study participants.

**Figure 3:**
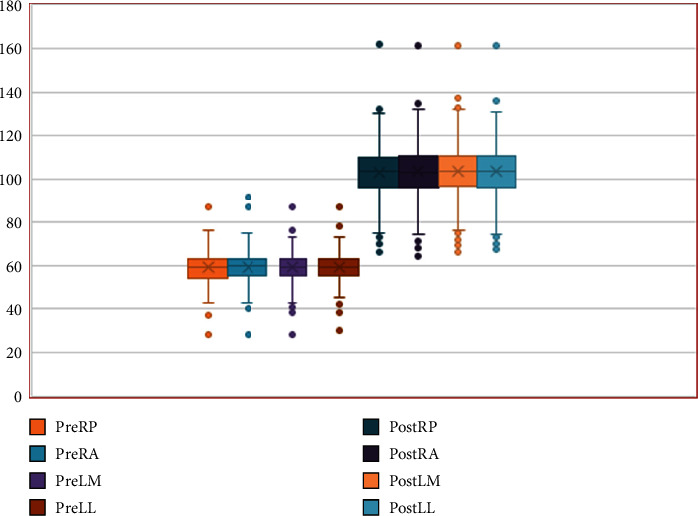
Box and Whisker plot illustrating the hepatic CT attenuations at the 4 locations in precontrast and portal venous scans. There is no significant variation in the hepatic attenuations at the locations, in both phases of the scans. RP = right posterior; RA = right anterior; LM = left medial; LL = left later.

There is no significant variation in the hepatic attenuation at the four different locations of measurement in both phases of the scans (Figures 1 and 3). The mean hepatic attenuation in the precontrast scan was 59.05 ± 6.69 HU while it was 103.73 ± 12.84 HU in the portal venous phase. The minimum and maximum recorded unenhanced hepatic attenuations were 28.7 HU and 89.80 HU, respectively (Table 1).

**Figure 4:**
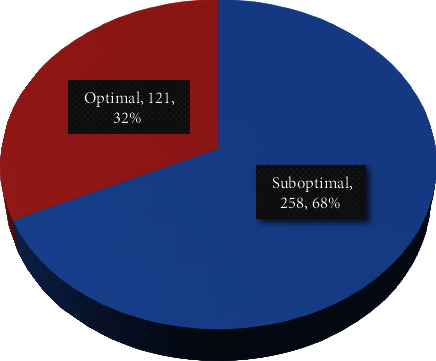
Contrast enhancement adequacy for patients with postcontrast abdominal CT at TASH with optimal defined as >50 HU increase from precontrast to portal venous phase, 2022 G.C.

**Figure 5:**
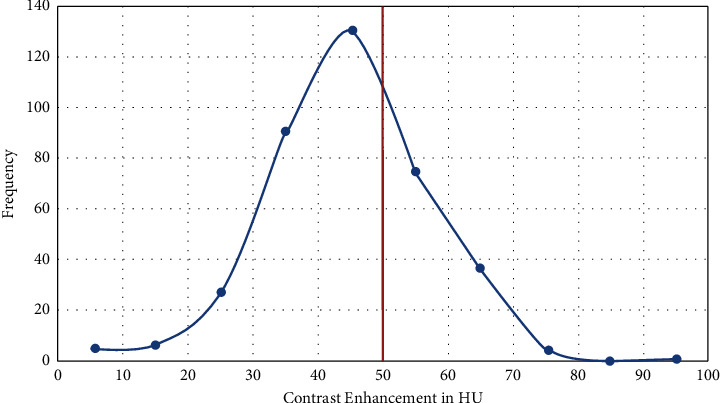
Contrast enhancement level distribution curve in CEIs, with the reference line at 50 HU, indicating the minimum required enhancement to label adequate, in patients with precontrast and postcontrast abdominopelvic CT performed via manual injection protocol at TASH, 2022 G.C.

**Figure 6:**
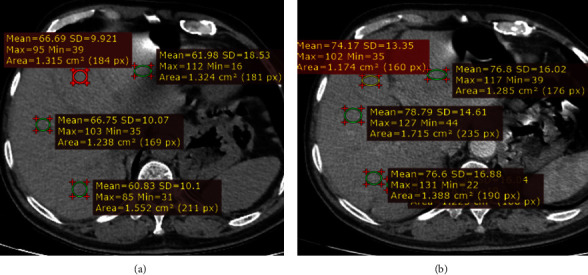
Axial precontrast (a) and postcontrast portal venous phase (b); axial CT scans at the same level taken in TASH, 2022 G.C, show mean hepatic attenuation at the four different locations of measurement. Mean hepatic attenuation was 64 HU on precontrast and 76.5 HU on PVP with CEI of 12.6 HU which is significantly lower than the expected minimum CEI of 50 HU.

**Figure 7:**
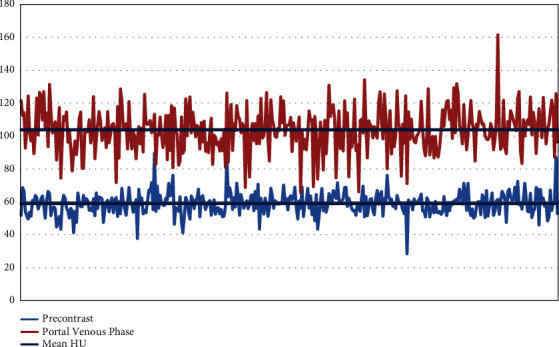
The precontrast and PVP hepatic attenuation values in abdominopelvic CT performed at TASH, 2022 G.C. Note there is wide variation in the portal venous line compared to the precontrast attenuation line with standard deviations of 12.8 HU and 6.7 HU, respectively.

The proportion of scans with less than 50 HU contrast enhancement was 68% (*n* = 258) which was below the level to label adequate enhancement, with only 32% attaining a value of more than 50 HU (Figure 4). There was wide variation in the level of contrast enhancement with CEIs ranging from 4.1 HU to 99.7 HU (Figure 5). This is illustrated in one of the axial CT scans in Figure 6. This is also reaffirmed by the high level of variation seen in the mean hepatic attenuation in the portal venous phase scan with a standard deviation of 12.97, while on the unenhanced scan, it was 6.69 HU (Figure 7). In addition, the ranges of the measurements were 60.9 HU on unenhanced scans and 95.65 HU on portal venous phase scans.

**Figure 8:**
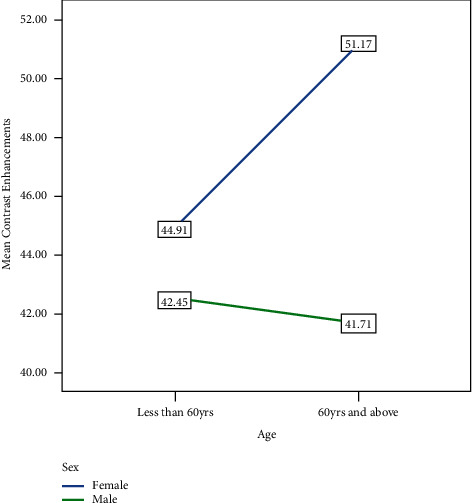
Mean contrast enhancements (CEIs) based on age and sex for patients evaluated with postcontrast abdominopelvic CT scans at TASH, Addis Ababa, Ethiopia, 2022 G.C.

The mean contrast enhancement index (CEI) in males and females was 42.26 ± 13.11 HU and 46.56 ± 12.39 HU, respectively. This difference in mean scores was statistically significant at a *P* value of 0.001. There were 97 (25.6%) patients aged 60 yrs and above. The mean CEI calculated was 43.82 ± 11.61 HU for those aged less than 60 years and 47.17 ± 15.77 years for those aged 60 years and above. However, this difference was not statistically significant with a *Pp* value of 0.057 (Figure 8).

Upon further analysis, the mean scores of contrast enhancement were calculated for those aged less than 60 years and those aged 60 years and above based on their sex categories. For those aged less than 60 years, the mean scores were 44.91 ± 10.52 in females and 42.45 ± 12.77 in males (*p* value = 0.077) and for those aged 60 years and above, the mean scores were 51.17 ± 15.75 in females and 41.71 ± 14.23 in males (*p* value = 0.003).

## 4. Discussion

The contrast enhancement pattern of abdominal CT at the study institution revealed that majority of the scans had inadequate contrast enhancement. This is evidenced by the significant proportion of suboptimal contrast enhancement index and the highly variable enhancement patterns across different patients.

The mean unenhanced liver attenuation in this study was 59.06 ± 6.69 HU. The density of an unenhanced normal liver at CT typically ranges between 55 and 65 HU [1]. In our study, the magnitude of hepatic parenchymal enhancement ranged from 4 to 99 HU with a mean of 44 ± 12 HU. The proportion of scans with optimal/adequate enhancement, which is defined by hepatic parenchymal enhancement 50 HU or more from the precontrast attenuation, was only 32%. These findings indicate that the precontrast attenuation measured in our study was relatively within the expected range. However, the hepatic enhancements measured at the portal venous phase are way below the expected range.

A study in Japan with two different CT scanners showed a precontrast hepatic attenuation of 60.8 ± 5.2 and 59.7 ± 6.0, which is within the expected normal range. The postcontrast CT hepatic attenuation on portal venous phase scan was 55.9 ± 9.6 and 62.9 ± 9.1, respectively, which is above the required standard, despite the significant differences in the enhancement between the two scanners.

There are several factors that affect the hepatic contrast enhancement pattern. The high rate of suboptimal hepatic enhancement in our setup could arise from the improper dosage as a fixed dose is administered for all patients irrespective of their total body weight or lean body weight. Yamashita et al. in a prospective randomized trial showed that contrast media administration tailored to the patient's weight resulted in more consistent organ enhancement and improved the image quality than those performed with fixed dosages [11].

Although the fixed dosage in our institution was in place in order to avoid the extra cost to patients if an additional vial of contrast is required, which is quite expensive in our setup, a retrospective cohort study of 6,737 subjects undergoing abdominal CT showed large cost and material savings can be realized by adopting a weight-based dosing strategy and lowering the maximum volume of administered contrast material [12].

Another reason could be the manual administration of contrast injection which affects the injection rate and injection duration despite the fact that this is proven to play fewer roles in portal venous phase scans compared to the arterial phase [7].

The female patients in this study had higher contrast enhancement than males with a mean difference of 4.3 HU. Other studies have also shown higher contrast enhancement in female patients than male patients with the administration of a fixed iodine load per body weight [7, 13]. This difference in magnitude can be explained by the lower blood volume in female patients than that of males with similar weight and height. In addition, since the lower blood volume leads to a higher blood flow rate for the same cardiac output, there would be earlier contrast enhancement in females than in males [7]. This difference in contrast enhancement based on sex was significantly more pronounced in those aged 60 years and above. This could be explained by the combined effects of the female sex and old age on blood volume and cardiac output [7].

In this study, the mean contrast enhancement of those aged 60 years and above was higher than those aged below 60 years, even though not statistically significant. One study indicated that elderly patients have a higher contrast enhancement for a given iodine load and suggested that iodine dose and injection rate could be reduced in elderly patients by 10% to achieve the same degree of enhancement [7, 14]. The possible explanations given for these include the reduction in blood volume and cardiac output with age. In addition, there is a slower excretion of contrast agents in elderly patients. Another explanation is the general pattern of weight change over the lifetime; weight grows up until about the age of 60 and then declines over the course of a lifetime [15].

## 5. Conclusion

The enhancement pattern of postcontrast abdominal CT at the study institution reveals a worrisome level of image quality. This is evidenced by the high number of suboptimal contrast enhancement indices and the highly variable enhancement patterns across different patients. This has a negative effect on the diagnostic performance of CT imaging which as a result may negatively impact management. Finally, age and sex need to be taken into consideration when administering contrast agents for imaging evaluation as these have been shown to have significant effects.

## 6. Recommendations

The contrast administration practice at the study institution needs to improve through the involvement of multiple stakeholders. Further study with contrast dose adjustment based on weight, age, and sex is recommended to see the effect of manual injection alone on CT studies and as such to evaluate its applicability in setups where an automatic injector is not available. Furthermore, future research studies in this area are recommended to assess for other causes of the high rate of suboptimal contrast enhancement and to determine the effect of diagnostic performance and patient management. The contrast agent dose administered should take into account the sex and age of patients.

## Figures and Tables

**Table 1:** Precontrast and portal venous phase hepatic attenuation measurements in patients with abdominal CT performed at TASH, 2020–2022 G.C.

	Precontrast	PVP	Enhancement index
Mean	59.05	103.73	44.68
Standard deviation	6.69	12.84	12.87
Minimum	28.70	65.63	4.1
Maximum	89.60	161.27	99.75
Range	60.9	95.65	95.65

## Data Availability

The data used to support the findings of the study can be obtained from the corresponding author upon request.

## Additional Points

This is a retrospective study and the study did not assess other possible factors that could affect the contrast enhancement pattern.

## References

[1] Boland G. W. L. (2014). *Gastrointestinal Imaging: The Requisites*.

[2] Brant W. (2019). *Fundamentals of Diagnostic Radiology*.

[3] Brunsing R. L., Fowler K. J., Yokoo T., Cunha G. M., Sirlin C. B., Marks R. M. (2020). Alternative approach of hepatocellular carcinoma surveillance: abbreviated MRI. *Hepatoma Research*.

[4] Kristie G., Louis H., Fred L., Dongqing W. (2013). Computed tomography in abdominal imaging: how to gain maximum diagnostic information at the lowest radiation dose. *Selected Topics on Computed Tomography*.

[5] van Cooten V. V., de Jong D. J., Wessels F. J., de Jong P. A., Kok M. (2021). Liver enhancement on computed tomography is suboptimal in patients with liver steatosis. *Journal of Personalized Medicine*.

[6] Taguchi N., Oda S., Nakaura T. (2018). Contrast enhancement in abdominal computed tomography: influence of photon energy of different scanners. *British Journal of Radiology*.

[7] Bae K. T. (2010). Intravenous contrast medium administration and scan timing at ct: considerations and approaches. *Radiology*.

[8] Zanardo M., Doniselli F. M., Esseridou A. (2018). Abdominal CT: a radiologist-driven adjustment of the dose of iodinated contrast agent approaches a calculation per lean body weight. *European Radiology Experimental*.

[9] Ichikawa T., Okada M., Kondo H. (2013). Recommended iodine dose for multiphasic contrast-enhanced mutidetector-row computed tomography imaging of liver for assessing hypervascular hepatocellular carcinoma: multicenter prospective study in 77 general hospitals in Japan. *Academic Radiology*.

[10] Manual A C R (2020). *On Contrast media*.

[11] George A., Manghat N., Hamilton M. (2016). Comparison between a fixed-dose contrast protocol and a weight-based contrast dosing protocol in abdominal CT. *Clinical Radiology*.

[12] Davenport M. S., Parikh K. R., Mayo-Smith W. W., Israel G. M., Brown R. K., Ellis J. H. (2017). Effect of fixed-volume and weight-based dosing regimens on the cost and volume of administered iodinated contrast material at abdominal CT. *Journal of the American College of Radiology*.

[13] Suzuki H., Oshima H., Shiraki N., Ikeya C., Shibamoto Y. (2004). Comparison of two contrast materials with different iodine concentrations in enhancing the density of the the aorta, portal vein and liver at multi-detector row CT: a randomized study. *European Radiology*.

[14] Itoh S., Ikeda M., Satake H., Ota T., Ishigaki T. (2006). The effect of patient age on contrast enhancement during CT of the pancreatobiliary region. *American Journal of Roentgenology*.

[15] Seidell J. C., Visscher T. L. (2000). Body weight and weight change and their health implications for the elderly. *European Journal of Clinical Nutrition*.

